# Sulfur signaling pathway in cardiovascular disease

**DOI:** 10.3389/fphar.2023.1303465

**Published:** 2023-11-24

**Authors:** Yunjia Song, Zihang Xu, Qing Zhong, Rong Zhang, Xutao Sun, Guozhen Chen

**Affiliations:** ^1^ Department of Pharmacology, School of Basic Medical Sciences, Heilongjiang University of Chinese Medicine, Harbin, China; ^2^ Department of Typhoid, School of Basic Medical Sciences, Heilongjiang University of Chinese Medicine, Harbin, China; ^3^ Department of Pediatrics, The Affiliated Yantai Yuhuangding Hospital of Qingdao University, Yantai, Shandong, China

**Keywords:** H_2_S, SO_2_, S-sulfhydration, S-sulfenylation, cardiovascular disease

## Abstract

Hydrogen sulfide (H_2_S) and sulfur dioxide (SO_2_), recognized as endogenous sulfur-containing gas signaling molecules, were the third and fourth molecules to be identified subsequent to nitric oxide and carbon monoxide (CO), and exerted diverse biological effects on the cardiovascular system. However, the exact mechanisms underlying the actions of H_2_S and SO_2_ have remained elusive until now. Recently, novel post-translational modifications known as S-sulfhydration and S-sulfenylation, induced by H_2_S and SO_2_ respectively, have been proposed. These modifications involve the chemical alteration of specific cysteine residues in target proteins through S-sulfhydration and S-sulfenylation, respectively. H_2_S induced S-sulfhydrylation can have a significant impact on various cellular processes such as cell survival, apoptosis, cell proliferation, metabolism, mitochondrial function, endoplasmic reticulum stress, vasodilation, anti-inflammatory response and oxidative stress in the cardiovascular system. Alternatively, S-sulfenylation caused by SO_2_ serves primarily to maintain vascular homeostasis. Additional research is warranted to explore the physiological function of proteins with specific cysteine sites, despite the considerable advancements in comprehending the role of H_2_S-induced S-sulfhydration and SO_2_-induced S-sulfenylation in the cardiovascular system. The primary objective of this review is to present a comprehensive examination of the function and potential mechanism of S-sulfhydration and S-sulfenylation in the cardiovascular system. Proteins that undergo S-sulfhydration and S-sulfenylation may serve as promising targets for therapeutic intervention and drug development in the cardiovascular system. This could potentially expedite the future development and utilization of drugs related to H_2_S and SO_2_.

## Introduction

**FIGURE 1:**
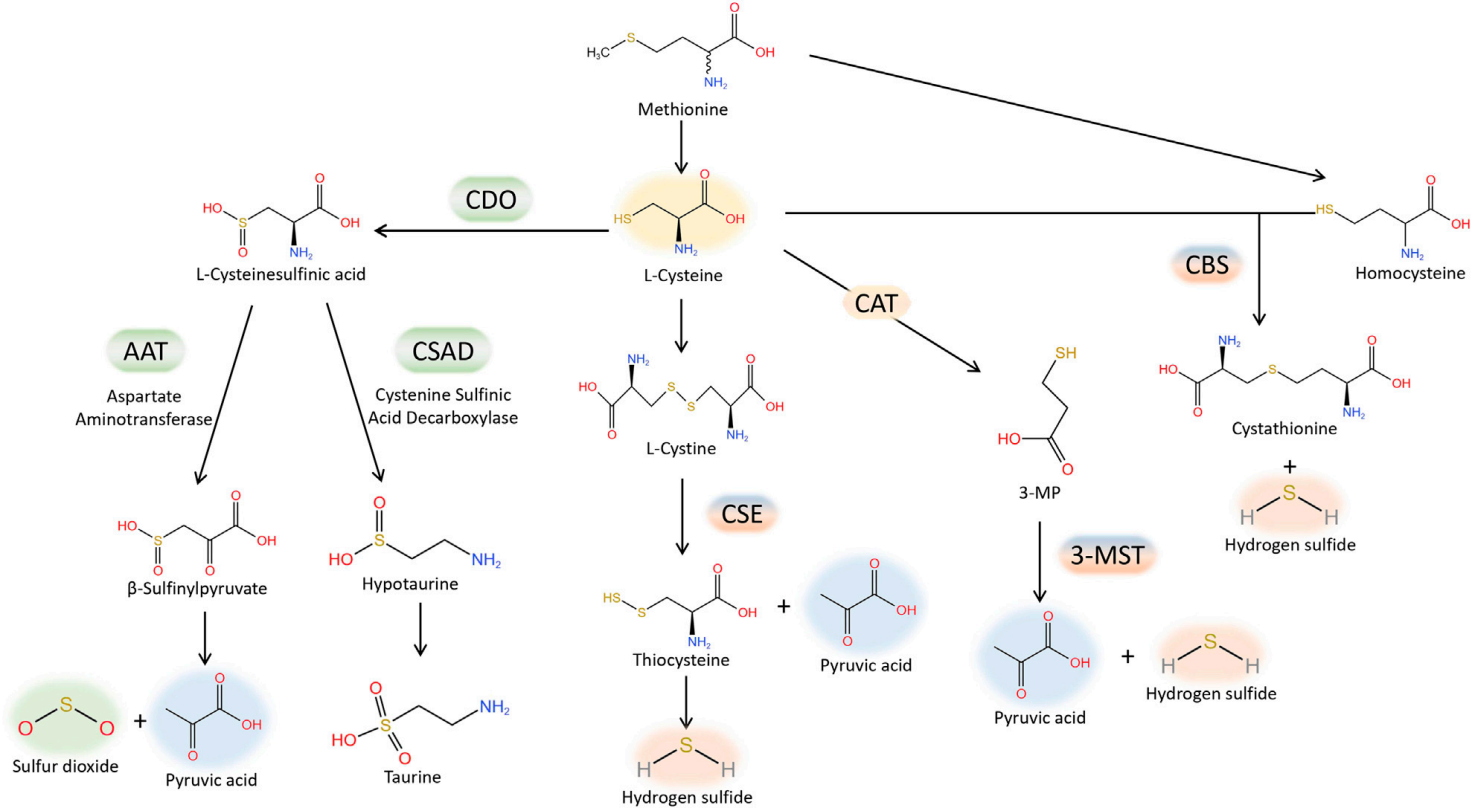
Generation of endogenous H_2_S and SO_2_.

H_2_S is regarded as the third gas signaling molecule, succeeding NO and CO. The production of H_2_S from L-cysteine is catalysed by cystathionine γ-lyase (CSE), cystathionine β synthase (CBS). Furthermore, H_2_S is also produced by 3-mercaptopyruvate sulfurtransferase (3-MST), which catalyzes the conversion of 3-mercaptopyruvate, generated by L-cysteine aminotransferase (CAT) from L-cysteine, into H_2_S. The production of H_2_S from L-cysteine is catalysed by cystathionine γ-lyase (CSE), cystathionine β synthase (CBS) and 3-mercaptopyruvate sulfurtransferase (3-MST). CSE is the primary enzyme responsible for producing H_2_S in the cardiovascular tissue (Banerjee et al., 2015). Lately, there has been an increasing amount of attention on SO_2_, which is closely related to H_2_S, within the cardiovascular system domain. Aspartate amino transferase (AAT) facilitates enzymatic reactions that convert sulfur-containing amino acids into SO_2_, utilizing L-cysteine as the substrate (Singer and Kearney, 1956). Interestingly, H_2_S and SO_2_ share tissue homology and originate from the same metabolic pathway (Figure 1). They exhibit comparable biological traits in cardiovascular physiological and pathological processes, including vasodilation, preservation of the typical vascular structure, and the development of conditions like pulmonary hypertension, atherosclerosis, endothelial dysfunction associated with aging, myocardial injury, and myocardial hypertrophy. As an illustration, it was discovered that H_2_S mitigated the harm to heart muscle cells caused by a lack of oxygen by diminishing the process of autophagy (Xiao et al., 2015); while in mice treated by AngII, it was demonstrated that SO_2_ inhibited autophagy, thereby attenuating cardiac hypertrophy as indicated by Chen et al. (Chen et al., 2016). Moreover, occasionally H_2_S and SO_2_, which are two gas signaling molecules, can utilize the identical signaling pathway. Activation of the PI3K/Akt pathway (Ji et al., 2016) can mediate protection against brain tissue ischemia-reperfusion (I/R) injury due to H_2_S. Additionally, the PI3K/Akt pathway plays a role in safeguarding against myocardial I/R injury caused by pretreatment with SO_2_ (Wang et al., 2011). Nevertheless, the precise workings of H_2_S and SO_2_ remain uncertain. Lately, an increasing number of scientists have discovered that certain impacts mentioned earlier could be ascribed to a new type of chemical alteration caused by H_2_S and SO_2_, referred to as S-sulfhydration, also named persulfidation, and S-sulfenylation. H_2_S or SO_2_ can chemically modify specific cysteine residues of target proteins through S-sulfhydration or S-sulfenylation, respectively. The thioredoxin system, closely associated with cardiovascular diseases (Li et al., 2023), reversed protein S-sulfhydration or S-sulfenylation, just like S-nitrosylation. The main focus of this review will be on the involvement of protein S-sulfhydration and S-sulfenylation by H_2_S and SO_2_ in the cardiovascular system.

## H_2_S induced protein S‐sulfhydration

**FIGURE 2:**
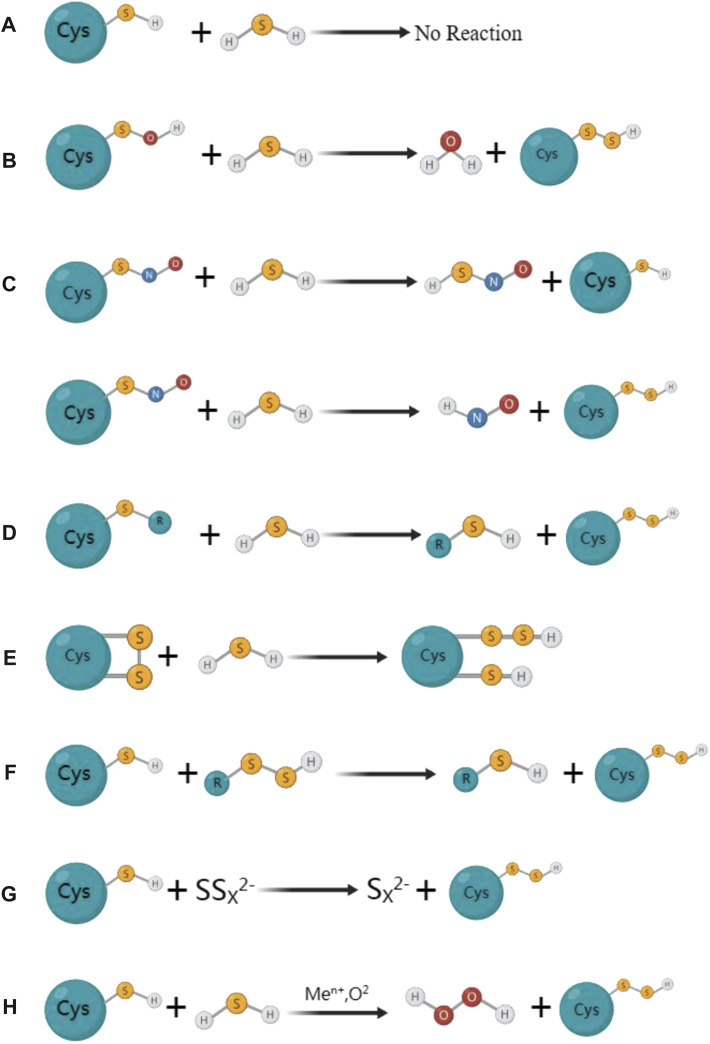
The mainly proposed formation processes for S-sulfhydrated proteins. **(A)** Protein sulfhydryl groups are not directly reacted with by H_2_S; **(B)** H_2_S can react with sulfinic acid to produce sulfhydryl groups; **(C)** H_2_S reacts with nitrosated cysteine to produce HSNO; However, depending on the protein environment, this reaction may also produce protein persulfides; **(D)** Persulfide can be created when H_2_S reacts with sulfur-containing molecules found in proteins; **(E)** While sulfhydryl can be created when H_2_S reacts with cysteine disulfide (-SS). **(F)** Persulfide can also be utilized as a carrier for the” trans-S-sulfhydration” reaction. **(G) (H)** Metal centers can act as oxidants and produce protein persulfides from H_2_S and thiolated proteins.

Thiolated proteins can be generated through S-sulfhydration, which is a common post-translational modification observed in approximatel one-third of proteins. The thiol modification of protein molecules is an essential molecular mechanism for H_2_S to exert various biological effects (Mustafa et al., 2009a; Paul and Snyder, 2012). Despite the growing fascination with protein S-sulfhydration, the exploration of mechanisms behind the formation of sulfhydrated proteins remains limited in the existing studies. Initially, it was believed that sulfhydryls on proteins could react directly with H_2_S to form protein persulfides, but this was a misconception. Due to thermodynamic limitations, the sulfhydryl group on the protein cannot directly react with H_2_S. During the S-sulfhydration, both sulfur atoms would be oxidised and gaseous hydrogen would be formed and disappeared. In this figure, we have demonstrated several primary processes of S-sulfhydrated modification, which may occur in the following scenarios: a) direct interaction between protein sulfhydryl groups and H_2_S is not observed; b) however, H_2_S has the ability to react with sulfinic acid and generate sulfhydryl groups; c) H_2_S reacts with nitrosated cysteine to produce HSNO; however, depending on the protein environment, this reaction may also produce protein persulfides; d) persulfide can be created when H_2_S reacts with sulfur-containing molecules found in proteins, e) while sulfhydryl can be created when H_2_S reacts with cysteine disulfide (-SS). f) persulfide can also be utilized as a carrier for the “trans-S-sulfhydration” reaction. g) and h), metal centers can act as oxidants and produce protein persulfides from H_2_S and thiolated proteins (Figure 2).

## Biological processes induced byS-sulfhydration

**FIGURE 3:**
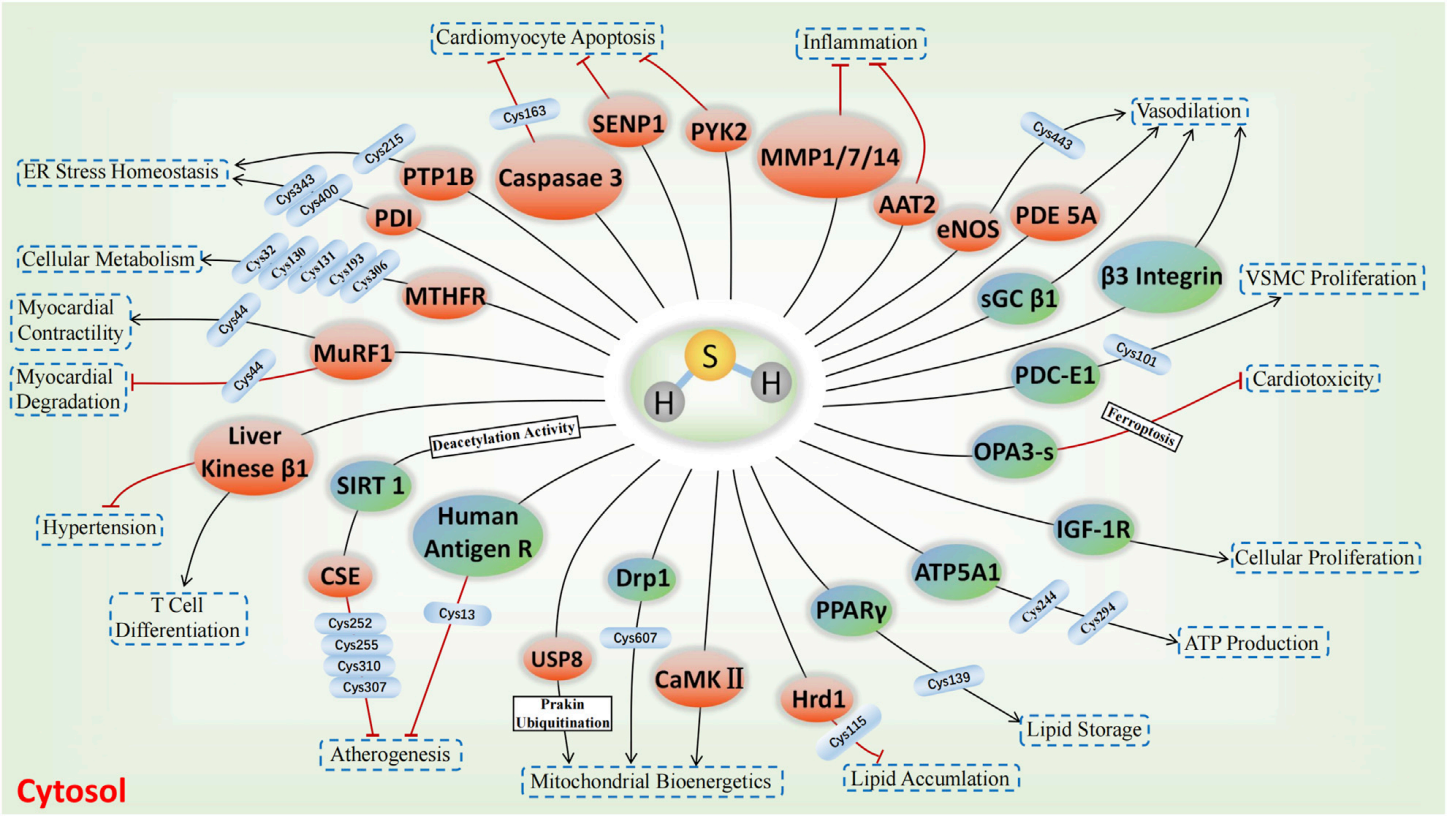
H_2_S-induced S-sulfhydrylation on enzymes and receptors in cardiovascular system. Orange means enzymes, and blue teal means receptors, → means stimulating effect, whereas ⟂ means inhibiting effect.

**FIGURE 4:**
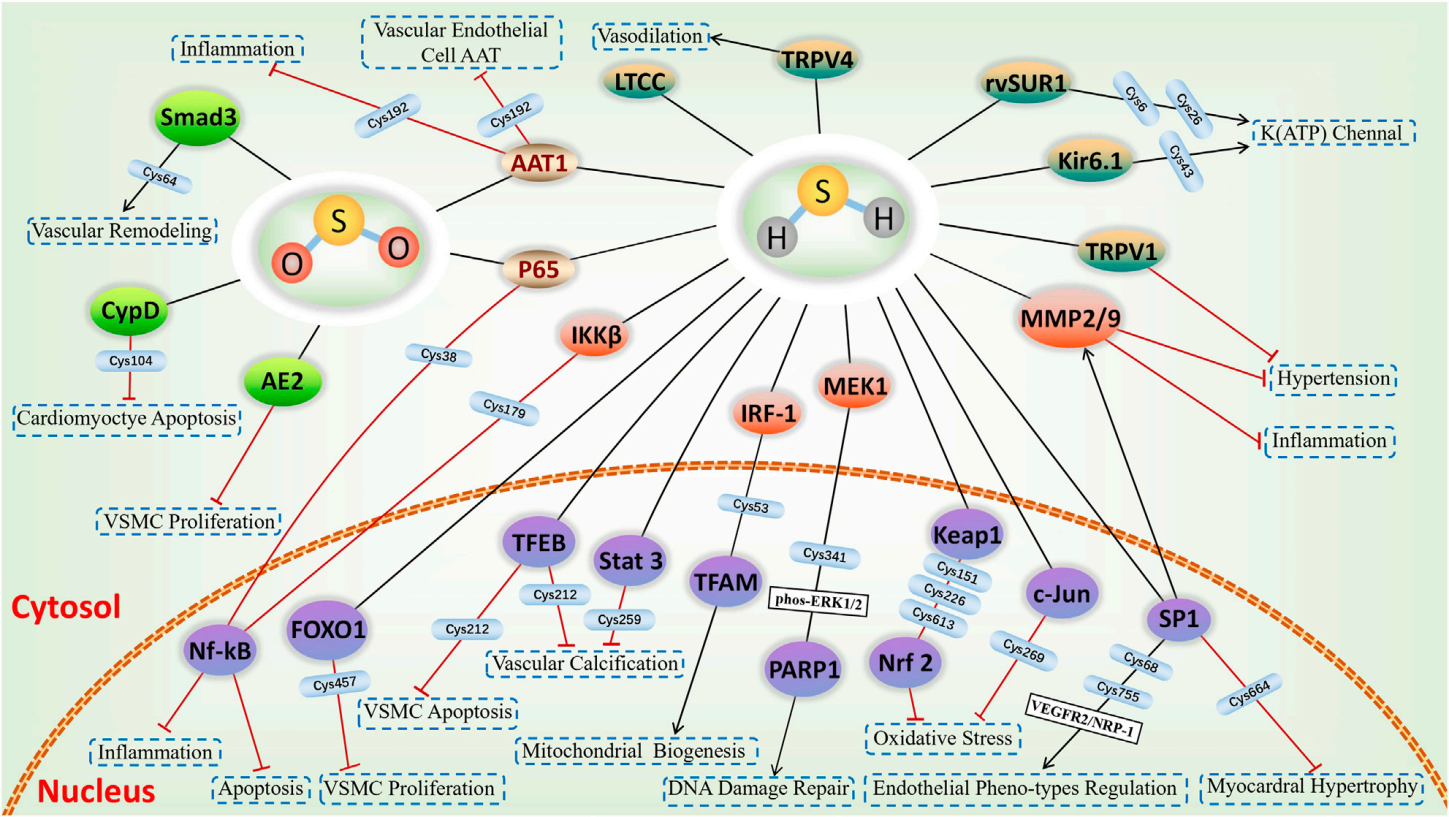
H_2_S-induced S-sulfhydrylation in ion channels and nuclear transcription factors, and SO_2_-induced S-sulfenylation in cardiovascular system. Yellow-green means ion channels, purple means nuclear transcription factors, green means targets of S-sulfenylation, and gold means common targets for S-sulfhydration and S-sulfenylation.

The involvement of sulfhydrated modification, a novel post-translational modification, in cardiovascular disease’s pathological processes is evident. Proteins undergo a transformation in activity and function after being S-sulfhydrated, playing crucial roles as significant toggles or controllers. We review some recent studies on the targets of S-sulfhydrated modification and explain the significant role of S-sulfhydration modification in various pathophysiological progression of the cardiovascular system (Figures 3, 4; Table 1).

**TABLE 1:** H_2_S-induced S-sulfhydraion on cardiovascular system.

Categories	S‐sulfhydrated proteins	Sites	Functions	Model	Reference
Enzymes	Caspase 3	Cys163	Anti-cardiomyocyte	DOX-treated H9c2	Ye et al. (2022)
			Apoptosis		
			Mitochondrial	TNF-α-treated HUVECs (*n* = 4)	Diaz et al. (2023)
			Bioenergetics		
	MEK1	Cys341	DNA damage repair	MMS-treated HUVECs	Zhao et al. (2014)
	PYK2		Anti-cardiomyocyte	AOAA-treated H9c2	Bibli et al. (2017)
			Apoptosis		
	MuRF1	Cys44	Myocardial contractility	db/db mice (*n* = 90)	Sun et al. (2020), Peng et al. (2022)
			Anti-myocardial	HG+Pal+Ole-treated NRCMs	
			Degradation		
	SENP1		Anti-cardiomyocyte	db/db mice (*n* = 50); HG+Pal+Ole-treated NRCMs	Peng et al. (2023)
			Apoptosis		
	Hrd1	Cys115	Lipid accumulation	db/db mice (*n* = 50); HG+Pal+Ole-treated NRCMs	Yu et al. (2020)
				db/db mice (*n* = 60); HG+Ole+Pal-treated H9c2	Sun et al. (2021)
		Cys32,Cys130,Cy			
	MTHFR	s131,Cys193,Cys	Cellular metabolism	High methionine diet-induced HHcy mice model (*n* = 10);	Ji et al. (2022)
		306		Hcy-treated HL-7702 cells and QSG-7701 cells	
	USP8		Mitochondrial	db/db mice (*n* = 50); HG+Ole+Pal-treated NRCMs	Sun et al. (2020)
			Bioenergetics		
	CaMKII		Mitochondrial	ISO-induced HF mice model; H_2_O_2_-treated H9c2	Wu et al. (2018)
			Bioenergetics		
	PTP1B	Cys215	ER stress homeostasis	Cardiomyocytes isolated from PTP1B-KO mice (C57BL/6J);	Kandadi et al. (2015), Coquerel et al. (2014), Kirshnan et al. (2011)
				Y615F-PERK mice; Tu and Tg treated-HEK-293T cells	
	PDI	Cys343,Cys400	ER stress homeostasis	Endothelial cell-specific CSE-KO or CSE-OE mice	Luo et al. (2023)
	IKKβ	Cys179	Anti-apoptosis	MCTP-treated HAPECs	Zhang et al. (2019)
	MMP1/7/14		Anti-inflammation		Zhu et al. (2022)
	MMP2/9		Anti-inflammation	SMCs isolated from CSE-KO mice; Human aneurysmal aortic samples	Zhu et al. (2022)
			Anti-hypertension		
	eNOS	Cys443	Vasodilation	AOAA and L-Cys-treated H9c2; AECs isolated from CSE-KO mice	Bibli et al. (2017), Altaany et al. (2014)
	PDE 5A		Vasodilation	NaHS or GYY4137-treated aortic rings isolated from rats (*n* = 8)	Sun et al. (2017)
	Liver kinase B1		Anti-hypertension	PBLs isolated from hypertensive patients and SHR	Cui et al. (2020)
	CSE	Cys252,Cys255,C	Anti-atherogenesis	Paigen and L-methionine induced ApoE-KO mice HHcy model (*n* = 45);	Fan et al. (2019)
		ys307,Cys310		L-homocysteine-treated HepG2 cells	
	AAT1		Anti-inflammation	CSE-knock down HUVECs, primary HUVECs and RPAECs	Zhang et al. (2018), Song et al. (2020)
	AAT2			MCT-induced male Wistar rats (*n* = 18)	
Receptors	PPARγ	Cys139	Lipid storage	HFD diet-induced obese mice model (*n* = 18);	Cai et al. (2016)
				IBMX, DEX and insulin-treated 3T3L1-preadipocytes	
	ATP5A1	Cys244,Cys294	ATP production	Deferoxamine and Nonidet-P40-treated HepG2 and HEK-293 cells	Módis et al. (2016)
				Male CSE-KO- C57/BL6 mice (*n* = 7)	
	Drp1	Cys607	Mitochondrial	TAC and ISO-treated C57BL/6 mice; CSE-KO mice (*n* = 9)	Wu et al. (2022)
			Bioenergetics		
	OPA3		Anti-cardiotoxicity	DOX-treated male C57BL/6 mice	Wang et al. (2023)
	IGF-1R		Cellular proliferation	IGF-1-treated SMCs isolated from CES-KO mice	Shuang et al. (2018, 2021)
	PDC-E1	Cys101	VSMC proliferation	db/db mice (*n* = 60); HG+Pal treated VSMCs	Zhang et al. (2021)
	sGC β1		Vasodilation	NaHS-treated rats aortic rings (*n* = 8)	Sun et al. (2017)
	β3 integrin		Vasodilation	Human LM, FG, FN and VN treated HUVECs; flow-treated ECs specific	Jalali et al. (2002), Bibli et al. (2021)
				CSE knockout mice	
	Human antigen R	Cys13	Anti-atherogenesis	Apolipoprotein -/- mice; Carotid plaques isolated from patients (*n* = 24)	Bibli et al. (2019)
	SIRT1		Anti-atherogenesis	ApoE-KO atherosclerosis mice (*n* = 20)	Du et al. (2019)
Ion channels	Kir 6.1 subunit of	Cys43	K(ATP) Chennal	Mesenteric arteries isolated from heparinized mice	Mustafa et al. (2011)
	KATP				
	rvSUR1 subunit of	Cys6,Cys26	K(ATP) Chennal	NEM and CLT-treated HEK-293 cells	Jiang et al. (2010)
	KATP				
	TRPV4		Vasodilation	Mesenteric arteries isolated from male SD rats; GSK1016790A-treated	Naik et al. (2016)
				AECs	
	TRPV1		Anti-hypertension	HA-induced WKY rats hypertension model; SHRs model (*n* = 8)	Yu et al. (2017)
	L-type calcium		Calcium channel	CaCl2 -treated A7r5 cells	Dai et al. (2019)
	(Ca2+) channels		opening		
Transcription factors	Sp1	Cys664	Anti-myocardral	Human myocardium samples of hypertension (*n* = 26); SHRs model	Meng et al. (2016)
			hypertrophy		
		Cys68,Cys755	Endothelial pheno-types	CBS-siRNA-transfected HUVECs (*n* = 3)	Saha et al. (2016)
			Regulation		
	IRF-1	Cys53	Mitochondrial	SMCs isolated from CSE-KO mice	Li et al. (2015)
			Bioenergetics		
	p65 subunit of	Cys38	Anti-inflammation	TNF-α-treated CSE-KO mice (*n* = 5); p65 C38S plasmid-transfected	Sen et al. (2012), Du et al. (2014), Zhang et al. (2019), Chen et al. (2017)
	NF-κB			THP-1-derived macrophages; MCTP-treated PAECs	
	c-Jun	Cys269	Anti-oxidative stress	H_2_O_2_-treated macrophage	Li et al. (2018)
	Keap1-Nrf2	Cys226,Cys613,C	Anti-oxidative stress	HS diet-treated Dahl rats (*n* = 30) and male SD rats (*n* = 40); STZ-treated	Yang et al. (2013), Hourihan et al. (2013), Huang et al. (2013), Xie et al. (2016)
		ys151,Cys273		Diabetes LDLr-/- mice (*n* = 6) and Nrf2-/- mice (*n* = 6)	
	FOXO1	Cys457	Anti-VSMC	ET-1-treated A7r5 and 293T cells	Tian et al. (2020)
			Proliferation		
	Stat3	Cys259	Anti-vascular	β-GP and ascorbate treated HASMCs	Zhou et al. (2019)
			Calcification		
	TFEB	Cys212	Anti-VSMC apoptosis	Human atherosclerotic plaque samples; VSMC-specific *cth* knockout	Chen et al. (2022)
			Anti-vascular	Mice; Autophagy inhibitor 3-MA and CQ-treated HASMCs	
			Calcification		

ASMCs, Artery smooth muscle cells; CLT, chloramine T; ET-1, endothelin-1; FG, fibrinogen; FN, fibronectin; HA, hydroxylamine; HAEC, Human aortic endothelial cells; HAPECs, human pulmonary artery endothelial cells; HASMCs, Human aortic smooth muscle cells; HEK-293, Human embryonic kidney cells; HepG2, Human hepatocellular carcinoma-derived cells; HG, high glucose; HS, high salt; HUVECs, Human umbilical vein endothelial cells; LM, laminin; MCTP, monocrotaline pyrrole; MEFs, Embryonic fibroblasts; MMS, methyl methanesulfonate; NEM, N-ethylmaleimide; NRCMs, Neonatal rat cardiomyocytes; Ole, oleate; PAEC, Pulmonary artery endothelial cell; Pal, palmitate; PBLs, Peripheral blood lymphocytes; PMA, 4β-phorbol-12-myristate-13-acetate; RPAECs, primary rat pulmonary artery endothelial cells; SHRs, Spontaneously hypertensive rats; STZ, streptozotocin; Tg, thapsigargin; Tu, tunicamycin; VN, vitronectin; WKY, Wistar‐Kyoto.

## H_2_S mediated S-sulfhydration on cardiovascular cell damage

The physiological process of apoptosis, also known as programmed cell death, is tightly controlled by cells or tissues for a variety of biological activities. Doxorubicin (DOX) is a potent anthracycline medication that effectively combats tumors. Nevertheless, it can induce apoptosis in cardiomyocytes, resulting in cardiotoxicity and influencing patients’ prognosis (Wenningmann et al., 2019). Cardiomyocyte apoptosis was significantly induced by DOX, leading to extensive activation of caspase family members. Apoptosis involves Caspase-3, which acts as a significant protease responsible for executing the process. A study from Ye et al. (Ye et al., 2022) uncovered that DOX diminished the CSE/H_2_S pathway, consequently leading to the apoptosis of cardiomyocytes. Additionally, enough endogenous H_2_S S-sulfhydration caspase-3 to block it from acting, reducing the apoptosis that DOX triggered in cardiomyocytes. Futher study found that the Cys-163 location of caspase-3 functioned as the specific site for H_2_S to sulfidate the caspase-3 protein. Diaz et al. (Diaz et al., 2023) discovered that H_2_S had the capability to reduce the mitochondrial redox condition, lower the activity of pro-caspase 3, and safeguard endothelial cells from apoptosis caused by TNF-α in isolation. Additionally, it was discovered that H_2_S increased the S-sulfhydration of pro-caspase 3 and enhanced the functioning of mitochondria in endothelial cells exposed to TNF-α. Furthermore, nuclear factor κB (NF-κB) functions as a transcription factor that inhibits apoptosis. In addition, the anti-apoptotic/pro-survival effects of H_2_S were attributed to the S-sulfhydration of NF-κB p65 (Sen et al., 2012). Nevertheless, the anti-cell death impact was nullified in macrophages derived from CSE^−/−^ mice, but it was reinstated through CSE overexpression or the addition of H_2_S. According to Sen et al., (Perkins, 2012), it was shown that H_2_S has the ability to alter NF-κB p65 at Cys38 thiol, augment the interaction between sulfhydrated p65 and its co-activator ribosomal protein S3, and stimulate the transcription of genes that prevent apoptosis. None of these effects were present following the transfection of p65-C38S. H_2_S additionally enhanced the S-sulfhydration of mitogen-activated extracellular signal-regulated kinase 1 (MEK1) in human endothelial cells (ECs) and human fibroblasts, whereas there was a reduced S-sulfhydration of MEK1 in CSE^−/−^ mice. MEK1 that has been sulfhydrated facilitates the phosphorylation of ERK1/2, which then moves into the nucleus to activate PARP-1, an abundant nuclear protein that plays a crucial role in DNA damage repair, and initiate the repair of DNA damage. Inhibition of ERK1/2 phosphorylation and PARP-1 activation, as well as the failure to facilitate DNA damage repair, were observed when Cys341 on MEK1 underwent mutation (Zhao et al., 2014).

Endothelial NO synthase (eNOS) is directly phosphorylated and inhibited by proline-rich tyrosine kinase 2 (PYK2), a tyrosine kinase that is sensitive to redox. A study from Bibli et al. (Bibli et al., 2017) found that when H9c2 cardiomyocytes were exposed to H_2_O_2_ or when H_2_S production was pharmacologically inhibited, there was an elevation in the phosphorylation of PYK2 (Y402) and eNOS (Y656). When Na_2_S was administered or CSE was overexpressed, these effects were blocked. The survival of H9c2 cells exposed to Η_2_Ο_2_ was diminished and further decreased following the suppression of H_2_S generation. These results suggest that H_2_S may alleviate the PYK2-mediated eNOS inhibition. Moreover, further studies revealed that the underlying mechanism was related to the S-sulfhydration modification of PYK2 and subsequent inhibition of its activity.

The primary adaptive response to cardiac hypertrophy occurs when cardiomyocytes encounter various damaging stimuli. Krüppel-like zinc-finger transcription factor 5 (KLF5), also known as BTEB2 and IKLF, played a crucial role in the progression of cardiac hypertrophy caused by angiotensin II (Shindo et al., 2002). A study by Meng et al. (Meng et al., 2016) discovered that in the cardiac tissues of hypertensive rats and angiotensin II treated cardiomyocytes, the H_2_S donor GYY4137 decreased the activity of the KLF5 promoter, lowered the level of KLF5 mRNA, hindered the transcriptional activity of KLF5, and consequently prevented the enlargement of heart cells. The aforementioned impacts of H_2_S were facilitated through its S-sulfhydration of specificity protein 1 (Sp1) at Cys664, causing Sp1 to be unable to bind to KLF5.

As a consequence of diabetes mellitus (DM), diabetic cardiomyopathy (DCM) causes anatomical and functional aberrancies in the myocardium, ultimately resulting in heart failure (HF). The presence of the cardiomyopathy is linked to elevated levels of the muscle RING finger-1 (MuRF1), which is an E3 ubiquitin ligase. A study from Sun et al. (Sun X. et al., 2020) demonstrated that H_2_S donor alleviated endoplasmic reticulum stress (ERS) in db/db mice, including the restoration of cardiomyocyte activity and structural repair. Additionally, H_2_S donor has the ability to inhibit the ubiquitination of myosin heavy chain 6 (MHC6) and myosin light chain 2 (MLC2) in the myocardial tissues of db/db mice. Subsequent investigation revealed that H_2_S S‐sulfhydrated MuRF1 at Cys44 to diminish its association between and MHC6 and MLC2, preventing myocardial degradation in the db/db mice. As a crucial calcium transport enzyme in the ER, SERCA2a has an impact on the relaxation and contraction of the myocardium. A study from Peng et al. (Peng et al., 2022) demonstrated that H_2_S donor effectively increased SERCA2a protein levels and activity, while decreasing its ubiquitination levels, as well as MuRF1 expression and cytosolic calcium concentrations in comparison to the db/db mice. Additional research revealed that the administration of NaHS increased the S-sulfhydration of MuRF1, subsequently boosting SERCA2a activity and expression. While, MuRF1-Cys44 mutant plasmid deteriorated H_2_S-mediated S-sulfhydration of MuRF1. The results indicated that H_2_S influences the ubiquitination of SERCA2a by S-sulfhydrating MuRF1 at Cys44, thereby preventing a decrease in myocardial contractility caused by elevated cytosolic calcium levels. Moreover, Peng et al. (2023) found that exogenous H_2_S suppresses SENP1s by S-sulfhydrating SENP1s at C683 site, which subsequently increases SERCA2asumo orylation, improves myocardial contractile-diastolic function, and reduces cardiomyocytes apoptosis in DCM.

## H_2_S mediated S-sulfhydration on cardiovascular cellular metabolism

Hyperhomocysteinemia (HHcy), an abnormal elevation of homocysteine in the plasma, hyperglycemia, and hyperlipidemia are recognized as risk factors resulting in various complications related to the cardiovascular diseases. The importance of H_2_S in regulating homocysteine, lipid, and glucose metabolism has been confirmed in numerous studies. CSE-H_2_S enhanced the nuclear accumulation of peroxisome proliferator activated receptor γ (PPARγ), its activity to bind DNA, and the expression of genes related to adipogenesis through directly S-sulfhydrating PPARγ at Cys139, resulting in the conversion of glucose into triglyceride storage within adipocytes. Based on what we know so far, PPAR has an important role in regulating blood lipid and glucose levels. Thereby, PPARγ S-sulfhydration could potentially serve as a new focus for addressing diabetes, obesity, hyperlipidemia, and associated cardiovascular complications (Cai et al., 2016).

HMG-CoA reductase degradation protein (Hrd1), an E3 ubiquitin ligase responsible for transiting protein. In the models of high glucose-treated db/db mice and neonatal rat cardiomyocytes, it was discovered that the levels of CSE and Hrd1 expression were reduced compared to the control mice, while CD36 and VAMP3 level was elevated. Further study found that administration of NaHS decreased the accumulation of lipids, restored the expression of Hrd1 as well as reduced the expression of VAMP3 and facilitated its ubiquitylation. The underlying mechanism is that H_2_S S-sulfhydrated Hrd1 at Cys115 to regulate VAMP3 ubiquitylation and prevent CD36 translocation in diabetic cardiomyopathy (Yu et al., 2020). Additionally, a study by Sun et al. (2021) demonstrated that the H_2_S donor could boost Hrd1 expression, as well as enhance DGAT 1 and 2 ubiquitination level in the myocardium of db/db mice. The underlying mechanism was associated with H_2_S-induced S-sulfhydration Hrd1 at Cys115, which boosted the connection between Hrd1 and DGAT1 and 2, ultimately preventing the development of liposome in the myocardial tissues of db/db mice.

The investigation of the key enzymes involved in Hcy metabolism is crucial as HHcy has been regarded as a contributing factor to cardiovascular disease. Methylenetetrahydrofolate reductase (MTHFR) is a pivotal enzyme controlling the Hcy metabolism within cells. A study from Ji et al. (2022) found that the bioactivity of MTHFR was decreased in HHcy of both vivo and vitro studies. The deficiency of H_2_S led to a further decrease in MTHFR activity and worsened HHcy. However, the decreased bioactivity of MTHFR in HHcy was reversed by H_2_S donors, resulting in a reduction of the excessive Hcy level. Furthermore, MTHFR undergoes H_2_S-mediated S-sulfhydration at Cys32, Cys130, Cys131, Cys193, and Cys306 in normal conditions, and the level of S-sulfhydration is directly linked to the bioactivity of MTHFR. The findings of this research indicated that H_2_S has the potential to enhance the bioactivity of MTHFR through S-sulfhydration, offering a potential therapeutic approach for HHcy.

## H_2_S mediated S-sulfhydration on cardiovascular mitochondrial bioenergetics

Over the past few years, mounting proof has indicated that H_2_S has the ability to preserve the structure of mitochondria, decrease the emission of signals indicating mitochondrial death, and mitigate cell death reactions regulated by mitochondria in different forms, thereby providing protection in the cardiovascular system (Szczesny et al., 2014). Under physiological conditions, H_2_S can cause a S-sulfydration of the α subunit of ATP synthase (ATP5A1) at Cys244 and Cys294. This process helps to sustain the activation of ATP synthase, thereby supporting mitochondrial bioenergetics (Módis et al., 2016). A study from Li and Yang, (2015) validated the significance of H_2_S in upholding the replication of mitochondrial DNA and the expression of genes that serve as markers for mitochondria. According to their findings, interferon regulatory factor 1 (IRF-1) was sulfhydrated at Cys 53 by H_2_S, which increased its affinity for the Dnmt3a promoter. This led to a decrease in DNA methyltransferase 3a (Dnmt3a) expression and the demethylation of the mitochondrial transcription factor A promoter, ultimately facilitating mitochondrial DNA replication. In addition, Wu et al. (2022) discovered that the CSE/H_2_S pathway regulates the activity and translocation of dynamin related protein 1 (Drp1), thereby influencing cardiac function and mitochondrial morphology. In terms of mechanism, H_2_S-mediated Drp1 S-sulfhydration at Cys607 caused a decrease in phosphorylation, oligomerization, and GTPase activity of Drp1, and directly competed with NO-mediated S-nitrosylation. This research revealed that H_2_S suppressed Drp1 activity through S-sulfhydrating Drp1 at Cys607, thereby protecting against HF.

DOX-induced cardiotoxicity is primarily attributed to ferroptosis a new type of cell death accompanied with an excessive amount of iron accumulation (Dixon et al., 2012). H_2_S had a defensive impact on DOX-triggered ferroptosis in cardiomyocytes according to the study from Wang et al. (2023). This effect was achieved through the involvement of optic atrophy 3 (OPA3), a crucial protein in the mitochondrial membrane. DOX caused a decrease in OPA3 levels, but exogenous H_2_S was able to restore them. OPA3 participates in the control of ferroptosis through its interaction with NFS1, resulting in the inhibition of ferroptosis. Exogenous H_2_S counteracted the ubiquitination of OPA3 induced by DOX through the promotion of OPA3 S-sulfhydration. These results indicated that H_2_S safeguards cardiomyocytes from DOX-induced ferroptosis by S-sulfhydrating OPA3, inhibiting the ubiquitination of OPA3 and enhances the expression of cysteine desulfurase (NFS1).

Mitochondrial injury caused by the excessive generation of reactive oxygen species (ROS) leads to myocardial injury in diabetic condition. A research by Sun Y. et al. (2020) discovered that H_2_S donor enhanced heart functions, decreased levels of reactive ROS, facilitated the movement of parkin into mitochondria, and stimulated the formation of mitophagy in the hearts of db/db mice. The aforementioned effects of H_2_S were associated with the rise in S-sulfhydration of USP8, resulting in the augmentation of parkin deubiquitination process by attracting parkin to mitochondria.

The involvement of Ca^2+^/calmodulin-dependent protein kinase II (CaMKII) is crucial in the progression of HF and the initiation of damage to myocardial mitochondria. In CSE knockout mouse models, it was discovered that administering H_2_S donor resulted in the mitigation of HF, decrease of lipid peroxidation, maintenance of mitochondrial function, and inhibition of CaMKII phosphorylation. And the potential mechanism could be associated with the S-sulfhydration of CaMKII by H_2_S, resulting in the inhibition of CAMKII activity and the maintenance of cardiovascular homeostasis (Wu et al., 2018).

## H_2_S induced S-sulfhydration on endoplasmic reticulum stress (ERS) in the cardiovascular system

The endoplasmic reticulum (ER) consists of a eukaryotic cell membrane and serves as a crucial organelle for the synthesis, folding, and secretion of proteins. ERS can be caused by changes in the external or internal environment. Numerous studies have shown that ERS is closely related to the onset and progress of various cardiovascular ailments. Protein tyrosine phosphatase 1B (PTP1B), a crucial player in ERS, is considered a promising candidate for therapeutic intervention in cardiovascular dysfunction caused by obesity and septic shock (Coquerel et al., 2014; Kandadi et al., 2015). Krishnan et al. (2011) discovered that H_2_S caused S-sulfhydration of PTP1B at Cys215, leading to the inhibition of its function. This inhibition, in turn, facilitated the phosphorylation and activation of protein kinase-like ER kinase, ultimately promoting the restoration of ER homeostasis. None of these effects were present in HeLa cells with CSE deletion. These results imply that H_2_S controls endoplasmic ERS by S-sulfhydration, leading to the deactivation of PTP1B. This could potentially serve as a new mechanism for the beneficial impact of H_2_S on the cardiovascular system.

Aortic aneurysm and aortic dissection (AAD) are serious conditions affecting blood vessels, where the primary focus of treatment for AAD is the endothelium. According to a research conducted by Luo et al. (2023), it was discovered that the deletion of CSE specifically in ECs worsened, while the overexpression of CSE specifically in ECs improved the advancement of AAD. During AAD, there was a decrease in S-sulfhydration of proteins in the endothelium, with protein disulfide isomerase (PDI) being the primary focus. Enhancing PDI activity and alleviating ERS was achieved through S-sulfhydration of PDI at Cys343 and Cys400. This data indicates that H_2_S mitigated the advancement of AAD by boosting the activity of protein PDI through the regulation of S-sulfhydration at Cys343 and Cys400 of PDI.

## H_2_S mediated S-sulfhydration on cardiovascular cellular inflammation

The connection between H_2_S and inflammation within the cardiovascular system is intricate. A study from Du et al. (Du et al., 2014) discovered that H_2_S suppressed the inflammation of macrophages caused by oxidized low-density lipoprotein through sulfhydrating NF-κB p65 at Cys38, which consequently inhibited the its phosphorylation, nuclear translocation and DNA binding activity. Furthermore, it was discovered that H_2_S suppressed macrophage inflammation caused by H_2_O_2_ through reducing the activation of the NLRP3 inflammasome, which resulted in the activation of caspase-1, ultimately decreasing the production of mitochondrial ROS (mtROS). The underlying mechanism is that H_2_S-induced S-sulfhydration of c-Jun increased transcriptional activity of SIRT3 and p62, leading to a reduction in mtROS production. Additional discoveries indicated that mutation of c-Jun Cys269 diminished the protection effects of H_2_S-induced c-Jun S-sulfhydration. To summarize, these findings indicate that H_2_S alleviates oxidative stress-mediated generation of ROS and the activation of the NLRP3 inflammasome in mitochondria through S-sulfhydration of c-Jun at Cys269 (Lin et al., 2018).

Inflammation of the ECs in the pulmonary artery is a crucial occurrence in the progression of pulmonary arterial hypertension (PAH). A study by Zhang et al. (Zhang et al., 2018) showed that in monocrotaline (MCT)-induced pulmonary vascular inflammation and CSE knockdown-induced ECs inflammation, H_2_S level was decrcased while SO_2_ level was increased. The underlying mechanism was related to the S-sulfhydration of AAT1/2 by H_2_S to inhibite the activity of AAT, leading to the reduction of endogenous SO_2_ generation. Additionally, the rise in endogenous SO_2_ production could potentially act as a compensative mechanism when the H_2_S/CSE pathway was suppressed, thus exerting protection against endothelial inflammatory responses. Furthermore, they showed that endogenous H_2_S effectively deactivated IKKβ by sulfhydrating Cys179 of IKKβ to suppress NF-κB pathway activation, ultimately attenuating pulmonary artery ECs inflammation in PAH (Zhang et al., 2019).

## H_2_S mediated S-sulfhydration on cardiovascular cellular oxidative stress

Oxidative stress is closely related to cardiovascular diseases. Several experimental findings indicate Keap1 and Nrf2 have a strong correlation with oxidative stress damage and antioxidant response. Nrf2 serves as a chief controller of the antioxidant reaction, while Keap1 functions as a suppressor of Nrf2 (Uesugi et al., 2017; Wasik et al., 2017). It was confirmed (Yang et al., 2013) that Keap1 underwent S-sulfhydration in embryonic fibroblasts obtained from mice with the WT genotype, whereas this modification was not observed in CSE knockout mice. In mouse embryonic fibroblasts, NaHS-induced S-sulfhydration of Keap1 at Cys151 to control Nrf2 expression, positioning and function. Possibly, this could be an innovative approach to hinder cellular aging through the S-sulfhydration of Keap1 mediated by H_2_S. Moreover, Hourihan et al. (Hourihan et al., 2013) additionally found that H_2_S deactivated Keap1 through the S-sulfhydration of Keap1 at the Cys226 and Cys613 location to upregulate the expression of Nrf 2, which subsequently protects cells from oxidative stress.

According to recent studies, H_2_S increased the S-sulfhydration of Keap1, leading to a decrease in the connection between Keap1 and Nrf2 in high-salt treated rat, which subsequently followed by a reduction in blood pressure, collagen buildup, and oxidative stress (Huang et al., 2016). The findings from aforementioned indicate that targeting H_2_S-induced S-sulfhydration of Keap1 could potentially help reduce oxidative stress and associated cardiovascular diseases.

## H_2_S induced S-sulfhydration on vascular structure

The excessive growth of vascular smooth muscle cells (VSMCs) serves as a crucial physiological and pathological foundation for numerous cardiovascular disorders. And H_2_S is discovered to maintain the structure of blood vessels by suppressing the proliferation of VSMCs. The receptor of insulin-like growth factor-1 (IGF-1), known as IGF-1R, has various effects on the vasculature, including promoting the growth and movement of VSMCs, as well as preventing the death of VSMCs both in normal and abnormal conditions. Studies from Shuang et al. found that H_2_S effectively reduces the levels of IGF-1R expression and promotes IGF-1R S-sulfhydration to weaken the interaction between IGF-1 and IGF-1R, elucidating the mechanism by which H_2_S inhibits VSMCs proliferation (Shuang et al., 2018; Shuang et al., 2021). Further study showed that H_2_S S-sulfhydrates IGF-1R to decrease formation of IGF-1R/ER-α hybrid, preventing estrogen-induced VSMCs proliferation (Shuang et al., 2021). In addition, a study from Tian et al. (Tian et al., 2020) found that the stimulation of ET-1 led to an augmentation in the proliferation of VSMC A7R5 cells, along with the phosphorylation of transcription factor forkhead box transcription factor 1 (FOXO1) and its subsequent relocation from the nucleus to the cytoplasm in the A7R5 cells. Nevertheless, administration of NaHS effectively nullified the aforementioned results induced by ET-1. Additionally, further study found that H_2_S hinders the phosphorylation of FOXO1 at Ser256 by S-sulfhydrating FOXO1 at Cys457. As a result, this action maintains the nuclear positioning and stimulation of FOXO1 while restraining VSMCs proliferation.

The proliferation of VSMCs induced by hyperglycaemia and hyperlipidaemia is inhibited by H_2_S. A study by Zhang et al. (Zhang et al., 2021) demonstrated that mitochondrial pyruvate dehydrogenase complex-E1 (PDC-E1) significantly translocated to the nucleus in VSMCs after high glucose and palmitate treatment. Further study found that H_2_S hindered the translocation of PDC-E1 through S-sulfhydration. Furthermore, PDC-E1 with a mutation at Cys101 abolished the inhibitory effect of H_2_S on the proliferation of VSMCs. These findings indicated that H_2_S prevented the translocation of PDC-E1 by S-sulfhydrating PDC-E1 at Cys101, subsequently inhibiting the proliferation of VSMCs treated with diabetic.

Insufficient growth of ECs is a crucial characteristic of endothelial dysfunction, leading to diseases related to vascular injury. The study according to Saha et al. (Saha et al., 2016) discovered that H_2_S derived from CBS preserved the cellular response dependent on VEGF, which includes proliferation induced by VEGF due to the upregulation of VEGFR-2 and neuropilin-1 in ECs. And the underlying mechanism was that H_2_S S-sulfhydrated the transcription factor Sp1 on Cys68 and Cys755 residues to enhance Sp1 binding to VEGFR-2, consequently boosting the proliferation and migration of ECs.

Maintaining elastin homeostasis is a crucial function of the CSE/H_2_S system. It was discovered that older CSE knockout mice experienced significant expansion of the aorta and deterioration of elasticity in the abdominal aorta, and exhibited heightened susceptibility to aortic elastic degradation induced by angiotensin II. While NaHS safeguarded against angiotensin II-induced aortic medial degeneration in old mice. Furthermore, application of NaHS or overexpression of CSE reduced the hyperactivity of MMP2/9 and elastolysis in TNFα-induced SMCs; however, CSE-deficiency worsened these effects. Additionally study discovered that H_2_S hindered the transcription of MMP2 through S-sulfhydrating Sp1. And H_2_S as well straightly inhibited excessive MMP activity through the S-sulfhydration of MMP1, MMP2, MMP7, MMP9, and MMP14. In sum, these results indicated that the CSE/H_2_S-induced S-sulfhydration, resulting in the inactivation of MMPs, contributes to the development of aortic elastolysis and medial degeneration (Zhu et al., 2022). This suggests that targeting the CSE/H_2_S system could be a potential treatment for aortic aneurysm.

Hyperglycemia can increase vascular calcification. The depletion of elastin in the tunica media encourages the SMCs to undergo an osteogenic transformation, leading to the calcification of arterial medial, which condition is linked to a significant cardiovascular risk in individuals diagnosed with type 2 diabetes. A study conducted by Zhou et al. (Zhou Y. B. et al., 2019) demonstrated that NaHS reduced the calcification of HASMCs exposed to high glucose by lowering levels of calcium and phosphorus, inhibiting calcium deposition and alkaline phosphatase (ALP) activity. Additionally, H_2_S hindered HASMCs osteogenic transformation by increasing the expression of SMα-actin and SM22α, which are two characteristic markers of smooth muscle cells, while decreasing the protein expression of core binding factor α-1 (Cbfα-1), a crucial factor in bone formation. Furthermore, the administration of NaHS suppressed the activation of Stat3, as well as the activity and expression of cathepsin S (CAS), while simultaneously elevating the elastin protein level. Further study found that inhibiting the activity or silencing the gene of Stat3 not only reversed the loss of elastin, but also reduced the expression of CAS. Elastin loss was alleviated by inhibiting CAS, whereas CAS overexpression worsened it. Additional research revealed that NaHS triggered S-sulfhydration in the wild type, but had no effect on the C259S Stat3 mutant. In conclusion, these findings indicate that H_2_S may directly S-sulfhydrated Stat3 at Cys259 and then inhibited Stat3/CAS signaling to upregulate elastin level, resulting in the attenuation of vascular calcification.

## H_2_S induced S-sulfhydration on vasorelaxtion

Vasorelaxation of H_2_S and its processes have been thoroughly researched as one of the significant physiologic activities caused by H_2_S. With the establishment of S-sulfhydration, a significant amount of knowledge has been gained regarding the molecular mechanisms underlying vasodilation induced by H_2_S.

H_2_S plays as a vasodilation by S-sulfhydration various KATP channels subunit. S-sulfhydration of Kir6.1, a component of the KATP channels, was observed upon overexpression of CSE, and this phenomenon was not observed in the absence or mutation of CSE. An additional investigation verified that S-sulfhydrated Kir6.1 at Cys43 reduced ATP synthesis while increasing the interaction between phosphatidylinositol 4,5-bisphosphate and Kir6.1, thereby enhancing KATP channel function and enhancing vasodilation. Furthermore, the Kir6.1-Cys43 mutants exhibited a reduction in both in S-sulfhydration and vasodilatation induced by H_2_S. Possibly, this could be the primary mechanism through which H_2_S functions as a relaxing factor derived from ECs (Mustafa et al., 2011). Furthermore, it was found that H_2_S-induced S-sulfhydration targeted Cys6 and Cys26 in rvSUR1, which is a subunit of the extracellular loop KATP channel complex in rats. The KATP channel was activated by H_2_S, leading to S-sulfhydration and subsequent relaxation of the blood vessels (Jiang et al., 2010). Additionally, Kang et al. (Kang et al., 2015) discovered that H_2_S S-sulfhydrated sulphonylurea 2B (SUR2B) at Cys24 and Cys1455, which are both part of the KATP channels complex, resulting in the recovery of smooth muscle contraction.

In previous studies, ECs are shown to produce endogenous H_2_S and to cause dilation in response to H_2_S. A study by Naik et al. (Naik et al., 2016) discovered that upon inhibiting TRPV4, the dilation of vessels caused by H_2_S-induced influx of Ca^2+^ and K^+^ was prevented. Furthermore, the S-sulfhydration of TRPV4 was increased following the administration of Na_2_S in aortic ECs. This implies that TRPV4 is triggered following S-sulfhydration, potentially serving as the crucial element in vasodilation. In addition, it was showed that the ability of the carotid sinus baroreceptor to regulate blood pressure was enhanced through the S-sulfhydration of TRPV1 by H_2_S derived from CBS, as indicated by Yu et al. (Yu et al., 2017). Additionaly, Dai et al. (Dai et al., 2019) discovered that NaHS decreased the level of intracellular Ca^2+^ by sulfhydrating L-type Ca^2+^ channels in VSMCs, thereby impacting the PKC/ERK pathway downstream and preventing the constriction of VSMCs.

The eNOS, an enzyme that produces NO, is a protein targeted by H_2_S, leading to vasodilation. A Study by Altaany et al. (Altaany et al., 2014) discovered that H_2_S enhances the activity of eNOS by causing the S-sulfhydration of eNOS at Cys443, which results in the promotion of eNOS phosphorylation and inhibition of its S-nitrosylation, ultimately leading to vasodilation. The soluble guanylatecyclase β1 (sGC β1), one of the subunits of the sGC protein, plays a crucial role as an enzyme in the process of catalyzing the synthesis of cGMP; on the other hand, phosphodiesterase (PDE) facilitates the breakdown of cGMP. And the sGC β1/PDE/cGMP is a signal transduction pathway associated with vascular relaxation. A study from Sun et al. (Sun et al., 2017) found that H_2_S increased cGMP synthesis by S-sulfhydrating sGC β1 and inhibited the degradation of cGMP by S-sulfhydrating PDE 5A to exert vasorelaxant effect in vascular tissues.

Integrins have been related to the detection of flow in ECs. The activation of β3 integrin occurred when shear stress was applied to ECs, causing a change in conformation (Jalali et al., 2002). A study from Bibli et al. (Bibli et al., 2021) discovered that the absence of S-sulfhydration hindered the connections between β3 integrin and Gα13, leading to the constant activation of RhoA and hindering the realignment of ECs caused by flow. Furthermore, there was a correlation between endothelial function and reduced H_2_S production, compromised dilation caused by flow, and the inability to detect β3 integrin S-sulfhydration. However, all of these results were restored when H_2_S supplement was administered. This study suggests that vascular illness is linked to significant alterations in the S-sulfhydration of proteins found in ECs, which play a role in facilitating responses to fluid movement. Enhancing vascular reactivity in humans was observed with the temporary addition of H_2_S, indicating the possibility of utilizing this pathway for the treatment of vascular disease.

Endogenous CSE/H_2_S in CD4^+^ T-cells plays an important role in the development of hypertension. In the case of hypertensive patients or spontaneously hypertensive rats, it was discovered that CSE/H_2_S in the isolated peripheral blood lymphocytes reacted to alterations in blood pressure. This was confirmed by variations in lymphocyte CSE protein and a negative association between H_2_S production and systolic and diastolic blood pressure. However, there was a positive association between H_2_S production and the interleukin 10 level of serum, which is an anti-inflammatory cytokine. The activation of liver kinase B1 by H_2_S derived from CSE, through constitutive S-sulfhydration, triggers the activation of its target kinase, AMP-activated protein kinase. This activation promotes the differentiation and proliferation of Treg cells, which helps to reduce immune-inflammation in the vascular and renal systems, ultimately preventing hypertension (Bibli et al., 2021).

## H_2_S induced S-sulfhydration on atherosclerotic

The presence of intimal plaques and cholesterol buildup in the arterial walls defines atherosclerosis, which is a primarily contributor to global mortality due to the susceptibility of plaque rupture. H_2_S, primarily produced by CSE in cardiovascular organs, serves as a safeguarding gasotransmitter in atherosclerosis (Zhang et al., 2013). A study from Chen et al. (Chen et al., 2022) found that CSE-H_2_S significantly decreased in ACTA2-positive cells within plaques from patients, atherosclerotic mice, or VSMCs stimulated with ox-LDL. And the H_2_S donor supplementation partially rescued the exacerbation of plaque size and reduction of autophagy, resulting from the deletion of CSE in VSMCs, thereby lowering plaque stability. In terms of mechanism, the S-sulfhydration of TFEB at the Cys212 site by CSE-H_2_S facilitates its translocation to the nucleus, subsequently enhancing VSMCs autophagy. This process promotes the secretion of collagen and suppresses apoptosis, ultimately reducing the progression of atherosclerosis and the vulnerability of plaques. Moreover, a study from Xie et al. (Xie et al., 2016) discovered that GYY4137 reduced the development of atherosclerotic plaques in the aorta and lowered levels of ROS in streptozotocin-induced LDL receptor knockout mice (LDLr^−/−^). However, this effect was not observed in mice with double knockout of LDLr^−/−^ and Nrf2^−/−^. GYY4137 additionally reduced foam cell development and oxidative stress in peritoneal macrophages obtained from wild type mice, while having no effect on Nrf2^−/−^ mice, implying that H_2_S mitigates the progression of atherosclerosis in diabetes through a mechanism that relies on Nrf2. Additional research revealed that GYY4137 facilitated the separation of Keap1 from Nrf2 in ECs stimulated by ox-LDL and high-glucose, potentially due to the S-sulfhydration of Keap1 at Cys151 and Cys273 sites. In the meantime, the Keap1 mutation at position C151A eliminated the dissociation of Keap1/Nrf2, the translocation of Nrf2 into the nucleus, and the inhibition of ROS induced by the administration of GYY4137. Therefore, it is suggested that the S-sulfhydration of proteins by H_2_S could serve as a new therapeutic objective for the prevention of atherosclerosis accelerated by diabetes. In addition, it was discovered that CSE specifically deficiency in ECs resulted in an increase in the expression of CD62E, which is associated with the activation of ECs and the development of atherosclerosis, and led to an elevated adherence of monocytes even without an inflammatory trigger, along with also accelerated the progression of endothelial dysfunction and atherosclerosis; but these effects were restored when treated with H_2_S donor. Mechanistically, the prevention of homodimerization and activity of human antigen R is achieved through the CSE-H_2_S induced S-sulfhydration at Cys13, leading to the attenuation of CD62E target protein expression (Bibli et al., 2019).

SIRT1, a crucial gene for promoting longevity, acts as a histone deacetylase and controls the acetylation of certain functional proteins, thereby exerting an anti-atherogenic impact. In atherosclerosis mice lacking ApoE, the administration of H_2_S donor, NaHS or GYY4137, resulted in decreased area of atherosclerotic plaque, infiltration of macrophages, inflammation in the aorta, and levels of lipids in the bloodstream. Treatment with H_2_S enhanced the expression of SIRT1 mRNA in the aorta and liver, as well as promoted SIRT1 deacetylation in ECs and macrophages, subsequently resulting in the reduction of inflammation in ECs and macrophages. Mechanismly, the direct S-sulfhydration of H_2_S on SIRT1 enhanced the binding of SIRT1 to zinc ion, subsequently boosting its deacetylation function and stability, ultimately reducing the formation of atherosclerotic plaques (Du et al., 2019).

Elevated levels of homocysteine can lead to various effects including dysfunction of the endothelium, heightened risk of blood clot formation, faster proliferation and movement of VSMCs, and hindered cholesterol transportation by monocytes and macrophages. These factors collectively contribute to the development of atherosclerosis (Thambyrajah and Townend, 2000; Lai and Kan, 2015). In the mice with atherosclerosis and hyperhomocysteinemia, it was discovered that the serum homocysteine level increased. Additionally, the mRNA, protein levels and catalytic activity of CSE, which is a crucial enzyme responsible for homocysteine trans-sulfuration, were reduced due to hyperhomocysteinemia; while the administration of H_2_S donor reversed all of these effects. In terms of mechanism, hyperhomocysteinemia caused S-nitrosylation of CSE, while H_2_S S-sulfhydrated CSE at the identical cysteine sites. Additional research revealed that the catalytic and binding capabilities of CSE towards L-homocysteine were reduced with S-nitrosylated CSE, while they were enhanced with S-sulfhydrated CSE. The alteration of Cys252, Cys255, Cys307, and Cys310 sites in CSE eliminated the S-nitrosylation or S-sulfhydration of CSE and hindered its interaction with L-homocysteine. To sum up, the administration of H_2_S donor improved the S-sulfhydration of CSE, leading to a reduction in serum levels of L-homocysteine. This, in turn, played a role in the beneficial effects against atherosclerosis observed in ApoE-knockout mice with hyperhomocysteinemia (Fan et al., 2019).

## SO_2_-induced S-sulfenylation on cardiovascular biological effects

Protein S-sulfenylation, also known as the oxidation of cysteine thiol to sulfenic acid (Cys-SOH), is a reversible post-translational modification, playing a pivotal role of SO_2_ in the modulation of the cardiovascular system (Figure 4). Following CO, NO, and H_2_S, endogenous SO_2_ has emerged as a new gas signalling molecule implicated in cardiovascular diseases. Hence, ensuring a consistent and appropriate production of endogenous SO_2_ is a crucial subject when it comes to maintaining cardiovascular balance. A study from Song et al. (Song et al., 2020) demonstrated that within vascular ECs, SO_2_ regulates its own production by employing negative feedback inhibition of AAT1 function through S-sulfenylation of Cys192 on AAT1. The discovery will significantly enhance the comprehension of regulatory mechanisms in maintaining cardiovascular balance.

According to recent research, it has been indicated that internal SO_2_ has the ability to alter different biological processes, including inflammation, apoptosis, as well as vascular remodeling. Moreover, it is suggested to have a therapeutic effect through S-sulfenylation. For example, SO_2_ induced S-sulfenylation of NF-κB p65 at Cys38, which resulted in the inhibition of NF-κB nuclear translocation and DNA binding activity. As a result, the NF-κB signaling pathway caused inflammation was inhibited, leading to a curative effect on oleic acid-induced acute lung injury (Chen et al., 2017).

The growth of cells relies on the pH level within the cells, known as intracellular pH (pHi). The alteration of cysteine in the transmembrane region of the Na^+^-independent Cl^−^/HCO_3_^−^exchanger, also known as anion exchanger (AE), has an impact on pHi. According to research conducted by Wang et al. (Wang et al., 2019) demonstrated that SO_2_ decreased the pHi and strongly activated AE. Conversely, the AE inhibitor greatly reduced the impact of SO_2_ on pHi in VSMCs. AE2 S-sulfenylation was associated with the impact of SO_2_. Moreover, the AE blocker abolished the inhibitory effect of SO_2_ on the proliferation of VSMCs stimulated by platelet-derived growth factor-BB (PDGF-BB). To summarize, this research showed that SO_2_ hinders the growth of VSMCs by directly activating the AE through posttranslational S-sulfenylation and causing intracellular acidification.

Another study by Huang et al. (Huang et al., 2021) determined SO_2_-induced S-sulfenylation proteomics through chemoproteomics in angiotensin II-treated VSMCs, which identified a total of 1137 S-sulfenylated cysteine residues in 658 proteins. Interestingly, 42% of these residues were found to be influenced by SO_2_. Among these, an increase in S-sulfenylation was detected in Cys64 of Smad3, resulting in a decrease in the ability to bind to DNA. Ultimately, the collagen protein levels were considerably inhibited, resulting in a reduction in angiotensin II-mediated vascular remodeling and abnormality.

Extended activation of mitochondrial permeability transition pore (mPTP) may result in impairment of mitochondrial energy, enlargement, breakage, programmed cell death, and necrosis (Zhou B. et al., 2019). Cyclophilin-D (CypD) serves as a significant controller in the modulation of mPTP opening (Sun et al., 2019). A study from Lv et al. (Lv et al., 2022) demonstrated that the SO_2_-induced S-sulfenylation of CypD at Cys104 leaded to the inhibition of mPTP opening, safeguarding cardiomyocyte against apoptosis.

## Detection of S-sulfhydration

**FIGURE 5:**
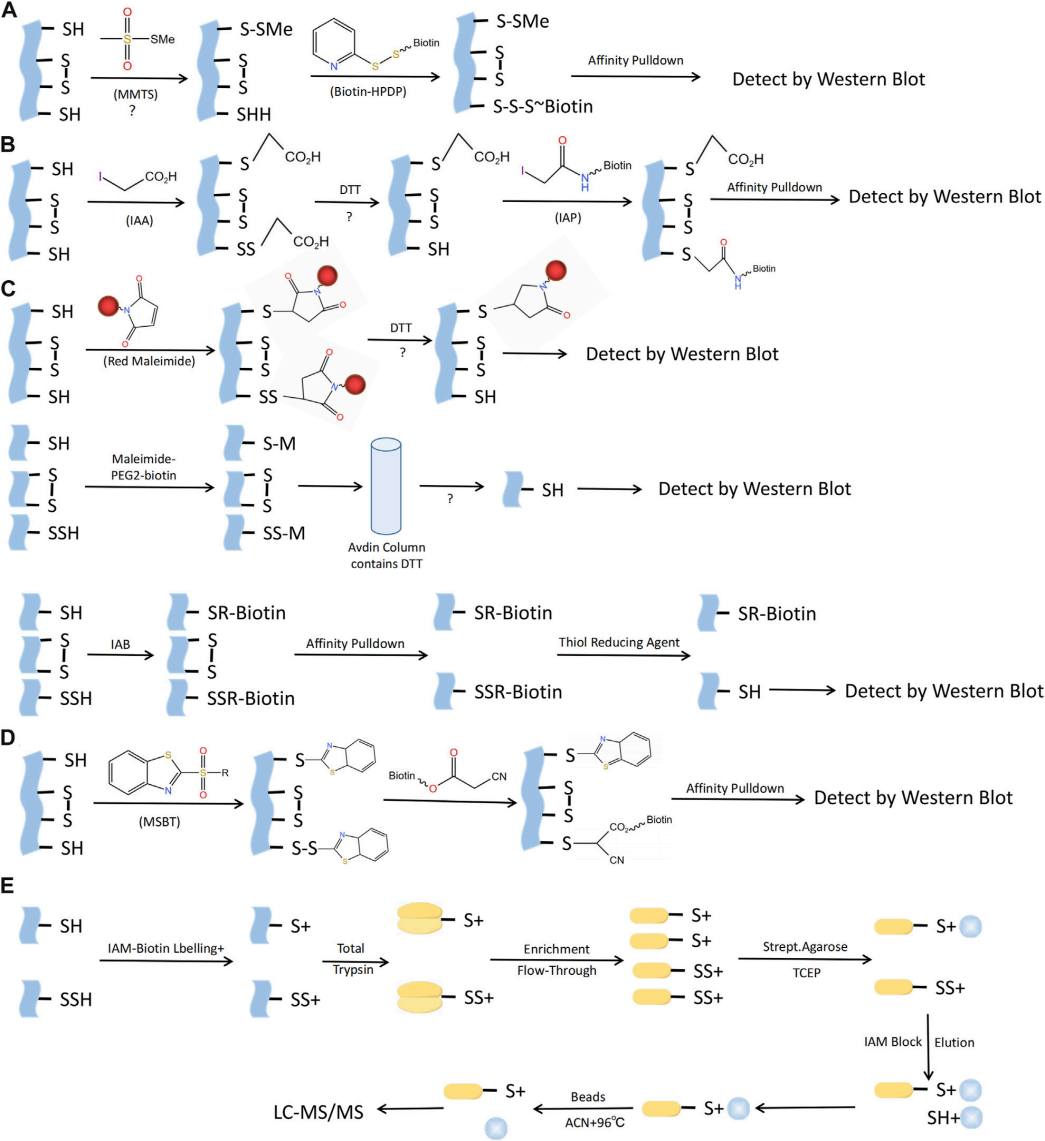
Methods for S-sulfhydraion detection. **(A)** Biotin-switch assay; **(B)** Cysteinyl labeling assay; **(C)** ➀ The maleimide assay, ➁Biotin-Thiol Assay, ➂ Protein persulfide detection protocol (ProPerDP); **(D)** Tag-switch assay; **(E)** Mass spectrometry assay. MMTS, S-methyl methanethiosulfonate; Biotin-HPDP, N-[6-(biotinamido)hexyl]-3’-(2′-pyridyldithio) propinamide; IAA, Iodoacetic acid; DTT, Dithiothreitol; IAP, Iodoacetamide-linked biotin; IAB, Iodoacetyl-PEG2-Biotin; MSBT, methylsulfonyl benzothiazole; IAM-Biotin, Iodoacetyl-PEG2-Biotin; TCEP, Tris (2-carboxyethyl)phosphine; IAM, Iodoacetamide; ACN, Acetonitrile; LC-MS/MS, Liquid chromatography and mass spectrometry.

There are several techniques for identifying S-sulfhydration, such as the altered biotin switch test, cysteinyl labeling test, maleimide test using fluorescent thiol modifying agents, tag-switch approach, and mass spectrometry (Figure 5). Nevertheless, currently there is no perfect technique for identifying S-sulfhydration due to the presence of inaccurate indications or inadequate sensitivity in the aforementioned methods. There is an urgent need for more specific methods to identify S-sulfhydration uniquely. An example of an original assay for detecting protein S-sulfhydration is the Biotin-Switch method (Mustafa et al., 2009b). The thiol in proteins was blocked by S-methyl methanethiosulfonate (MMTS), an alkylating agent. Subsequently, Biotin-HPDP was conjugated with the persulfides group. Nevertheless, this approach facilitated the concurrent labeling of S-sulfhydration and S-nitrosylation, resulting in poor selectivity. The cysteine labeling method uses IAA as a blocking agent, and IAP is used to label the persulfide modified proteins (Krishnan et al., 2011). One concern with this approach is its inability to differentiate persulfides from intramolecular, intermolecular, and S-nitrosothiols, all of which will also be broken down by DTT. The maleimide test relies on the chemical properties of N-ethyl maleimide, a reagent that blocks both free thiol and persulfide groups (Sen et al., 2012). A drawback of this fluorescence technique is its limited applicability for proteomic analysis. The Biotin-Thiol-Assay can employ NM-Biotin or IAB to alkylate both thiol and persulfide functional groups (Gao et al., 2015; Dóka et al., 2016), but this approach may result in inaccurate negative signals.

Considering the aforementioned issues with the Biotin-Switch technique and maleimide approach, Zhang et al. proposed the tag-switch assay to detect S‐sulfhydration modification, the core of which is the use of two different reagents to label supersulfide. Currently, the eligible thiol sealers are methansulfonyl benzothiazole (methylsulfonylbenzothiazole, MSBT) and methyl cyanoacetate (Park et al., 2015; Wedmann et al., 2016). Furthermore, the analysis of protein S‐sulfhydration also involved the utilization of mass spectrometry (MS). By obstructing sulfol groups in the proteins using MSBT, the biotin-labeled proteins were subsequently separated into polypeptides in order to detect persulfated modified proteins and their respective locations. Nonetheless, this method presents an equal challenge in fully obstructing protein samples and, as a result, can easily produce inaccurate positive outcomes (Park et al., 2015).

To summarize, the exploration of S‐sulfhydration alteration is still in its early stages, and the criticality of choosing exceptionally precise detection agents cannot be overstated in advancing this domain. Furthermore, the integration of the aforementioned testing technique with mass spectrometry can effectively prevent inaccurate positive outcomes. In addition, the development of fluorescent probes that detect S-Sulfhydrylation protein imaging, even commercially available ones, is also worthwhile. In short, the exploration of the detection methods for S‐sulfhydration modification will provide an insight into the biological significance of this post-translational modification.

## Detection of S-sulfenylation

**FIGURE 6:**
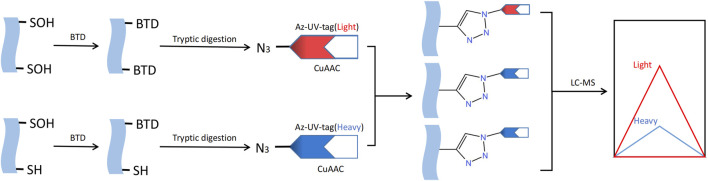
Methods for S-sulfenylation detection. S- sulfenylated proteins in cells treated are labeled with the BTD. Labeled proteins are further conjugated with light and heavy azido biotin with a photocleavable linker and analyzed by LC-MS/MS.

S-sulfenylation, a post-translational modification that can be reversed, is crucial for regulating protein activity through redox control in numerous biological processes. The detection and study of protein S-sulfenylation is not possible directly because it is inherently unstable. Over the last few decades, different dimedones (aka dicarbonyl) are now more readily available for the specific identification and detection of cysteine S-sulfenylation (Furdui and Poole, 2014). For instance, Western blotting with the appropriate antibody can be employed to detect cysteine S-sulfenylation labeled with dimedone (Seo and Carroll, 2009). Dimedone analogs containing fluorescent or biotin reporter groups can be used to visualize and enhance S-sulfenylated proteins (Charles et al., 2007). Carroll Lab created the initial DAz-1 probe for detecting sulfenic acid in its natural environment. This compound is dimedone that has been chemically modified with an azide group, enabling its selective recognition by phosphine reagents through the Staudinger ligation method. This technique is used for the detection, enrichment, and visualization of altered proteins (Reddie and Carroll, 2008). In 2012, the Carroll laboratory developed DYn-2, a novel dimedone analog labeled with alkyne that had superior stability and efficiency compared to previous probes based on dimedone for labeling Cys-SOH *in situ* (Paulsen et al., 2011). The use of dimedone-based probes has greatly expanded the number of S-sulfenylated proteins and their corresponding sites. Several other chemical compounds, apart from dimedone, have been extensively studied for the specific labeling of S-sulfenic acids (Qian et al., 2012; Poole et al., 2014; McGarry et al., 2016; Alcock et al., 2018). In order to develop the next iteration of chemoproteomic probe for the worldwide exploration of S-sulfenylome, Carroll Lab initially constructed an innovative collection of 100 cyclic carbon-nucleophiles that selectively interact with Cys-SOH (Gupta and Carroll, 2016). Expanding on this source, they additionally created four novel alkyne-labeled probes, namely, TD, PYD, PRD, and BTD, for the specific marking of protein S-sulfenic acids. Due to its exceptional response rate towards Cys-SOH, BTD displayed the utmost degree of reactivity towards S-sulfenylome (Gupta et al., 2017). And BTD has demonstrated a strong compatibility with chemoproteomic platforms that focus on specific sites. Hence, the novel BTD probe (Fu et al., 2019) can be utilized to achieve a more efficient approach in mapping and quantifying cysteine S-sulfenylation in intricate proteomes (Figure 6).

## Conclusion

Despite the notable advancements in drug treatment and clinical guidance for cardiovascular diseases, the prevalence and fatality rates of such conditions persist at elevated levels due to the aging population and escalating risk factors. Consequently, there is an urgent demand for novel therapeutic concepts and strategies to address cardiovascular diseases. In this context, the discovery of H_2_S and SO_2_ as gas signaling molecules in recent years has emerged as a significant development, as they exhibit crucial protective effects within the cardiovascular system. Currently, there is ongoing development of various H_2_S and SO_2_ donors or targeted prodrugs. In recent years, different types of SO_2_ donors and prodrugs with distinct triggering mechanisms have been designed, including thiol-activated SO_2_ prodrugs (Zhang et al., 2023), thermally-activated SO_2_ prodrugs (Li et al., 2019), hydrolysis-based SO_2_ prodrugs (Day et al., 2016), glutathione-responsive SO_2_ prodrugs (Xia et al., 2022), and esterase-sensitive SO_2_ prodrugs (Wang and Wang, 2017). Additionally, H_2_S donors such as CySSPe (Tocmo and Parkin, 2019) and Diallyl trisulfide (Qiao et al., 2018), and the mitochondrial targeting of H_2_S prodrugs AP39 and RT01 (Magierowska et al., 2022), as well as photothermal therapy-triggered H_2_S prodrugs (Chen et al., 2015), have emerged as novel strategies for the treatment of cardiovascular diseases. Over the last decade, an increasing number of studies have elucidated the diverse biological regulatory functions of H_2_S and SO_2_, specifically through the direct S-sulfhydration and S-sulfenylation of target proteins. These modifications have been shown to effectively and promptly regulate cell signal transmission. Notably, significant progress has been made in comprehending the role of protein S-sulfhydration and S-sulfenylation mediated by H_2_S and SO_2_ in the cardiovascular system. It is undeniable that research on protein S-sulfhydration and S-sulfenylation is being increasingly suggested as a prospective avenue for future investigations in the realm of gas signaling molecules. Consequently, the exploration and creation of cardiovascular protective medications that target S-sulfhydration and S-sulfenylation may represent a novel path for clinical drug treatment of cardiovascular injury diseases. In light of this, it is imperative to collaborate with the fields of drug research and development and pharmacology research to facilitate the translation of fundamental research into clinical applications.

However, there exist numerous significant concerns pertaining to the utilization of H_2_S-induced S-sulfhydration and SO_2_-induced S-sulfenylation in drug development, which necessitate attention for their prospective clinical application. (1) During the protein S-sulfhydration process, the generation of both small-molecule based persulfides and protein persulfides occurs, resulting in highly reactive species. The metabolic regulation of these species remains largely unexplored. (2) it is intriguing to investigate the distinct utilization of H_2_S and SO_2_ by cardiovascular cells at specific temporal intervals. (3) There is an urgent requirement for improved scientific techniques that possess greater sensitivity and specificity in order to identify S-sulfhydration. (4) Further research is required to explore additional proteins and thoroughly examine the specific cysteine sites associated with S-sulfhydration and S-sulfenylation within the cardiovascular system. (5) Nevertheless, not every protein that undergoes S-sulfhydration and S-sulfenylation experiences a modified spatial arrangement and functionality. The determination of this could depend on the positioning of the cysteines that are S-sulfhydrated/S-sulfenylated. Protein function and signal transduction will be altered if S-sulfhydrated/S-sulfenylated cysteines are found in the crucial domain, which is essential for maintaining the structure and activity of the protein. Put simply, there could be no notable distinction following S-sulfhydration and S-sulfenylation, commonly referred to as ‘ineffective S-sulfhydration and S-sulfenylation’. (6) Furthermore, further studies will explore the importance of S-sulfhydration/S-sulfenylation in the cardiovascular system, including but not limited to target gene transcription, enzymatic activity, and ion channel permeability. (7) The thioredoxin system regulates the levels of S-sulfhydration and S-sulfenylation, indicating that modifying the activity or expression of thioredoxin may play a role in controlling the intracellular levels of the two modifications and the biological and pharmacological effects mediated by H_2_S and SO_2_. (8) Further investigation is warranted to explore the potential interactions between S-sulfhydration and S-sulfenylation and other post-translational modifications, with the aim of expediting the advancement of cardiovascular disease treatment. (9) A comprehensive examination is necessary to thoroughly explore the clinical significance of S-sulfhydration and S-sulfenylation in cardiovascular disorders. (10) Additionally, it is important to acknowledge that proteins modified through S-sulfhydration and S-sulfenylation may elicit biological effects by activating downstream components of the target protein. For instance, the anti-oxidation effect of Keap1 modified by H_2_S can be observed in the activation of Nrf2 in the Keap1-Nrf2 pathway, leading to the activation of downstream anti-oxidation genes. However, it is important to note that the activation of Nrf2 is not solely regulated by Keap1, and excessive Nrf2 activation can result in bodily abnormalities. Therefore, the control of drug release is crucial in minimizing adverse reactions.

Protein S-sulfhydration or S-sulfenylation, a crucial post-translational modification induced by H_2_S or SO_2_, may potentially function as a molecular mechanism underlying the effects of H_2_S or SO_2_. Further exploration is necessary to determine the clinical significance of S-sulfhydration and S-sulfenylation in cardiovascular disorders. Acquiring additional knowledge concerning S-sulfhydration and S-sulfenylation will augment our understanding of the beneficial influence that these modifications can exert on specific cysteines in various cardiovascular conditions. Furthermore, the proteins that are S-sulfhydrated and S-sulfenylated could serve as promising new targets for therapeutic intervention and drug development in the cardiovascular system. This, in turn, could expedite the advancement and utilization of drugs associated with H_2_S or SO_2_ in the coming years.
